# Dynamic Changes in Amino Acid Concentration Profiles in Patients with Sepsis

**DOI:** 10.1371/journal.pone.0121933

**Published:** 2015-04-07

**Authors:** Longxiang Su, Hua Li, Aimei Xie, Dan Liu, Weiqiao Rao, Liping Lan, Xuan Li, Fang Li, Kun Xiao, Huijuan Wang, Peng Yan, Xin Li, Lixin Xie

**Affiliations:** 1 Department of Respiratory Diseases, Chinese PLA General Hospital, Beijing, China; 2 Department of Critical Care Medicine, Peking Union Medical College Hospital, Peking Union Medical College & Chinese Academy of Medical Sciences, Beijing, China; 3 Clinical Metabolomics Platform, BGI Health, BGI Shenzhen, Shenzhen, China; 4 Medical School, Nankai University, Tianjin, China; 5 Clinical Division of Internal Medicine, Chinese PLA General Hospital, Beijing, China; Rosalind Franklin University, UNITED STATES

## Abstract

**Objectives:**

The goal of this work was to explore the dynamic concentration profiles of 42 amino acids and the significance of these profiles in relation to sepsis, with the aim of providing guidance for clinical therapies.

**Methods:**

Thirty-five critically ill patients with sepsis were included. These patients were further divided into sepsis (12 cases) and severe sepsis (23 cases) groups or survivor (20 cases) and non-survivor (15 cases) groups. Serum samples from the patients were collected on days 1, 3, 5, 7, 10, and 14 following intensive care unit (ICU) admission, and the serum concentrations of 42 amino acids were measured.

**Results:**

The metabolic spectrum of the amino acids changed dramatically in patients with sepsis. As the disease progressed further or with poor prognosis, the levels of the different amino acids gradually increased, decreased, or fluctuated over time. The concentrations of sulfur-containing amino acids (SAAs), especially taurine, decreased significantly as the severity of sepsis worsened or with poor prognosis of the patient. The serum concentrations of SAAs, especially taurine, exhibited weak negative correlations with the Sequential Organ Failure Assessment (SOFA) (r=-0.319) and Acute Physiology and Chronic Health Evaluation (APACHE) II (r=-0.325) scores. The areas under the receiver operating characteristic curves of cystine, taurine, and SAA levels and the SOFA and APACHE II scores, which denoted disease prognosis, were 0.623, 0.674, 0.678, 0.86, and 0.857, respectively.

**Conclusions:**

Critically ill patients with disorders of amino acid metabolism, especially of SAAs such as cystine and taurine, may provide an indicator of the need for the nutritional support of sepsis in the clinic.

**Trial Registration:**

ClinicalTrial.gov identifier NCT01818830.

## Introduction

The metabolic disorders of critical illnesses are highly complex [1]. Sepsis, one such illness, is characterized by high morbidity and high mortality [2] and is often accompanied by significant metabolic changes such as metabolic hyperactivity, a high catabolic state, increased resting energy expenditure, increased protein catabolism, increased fat catabolism, negative nitrogen balance, insulin resistance, hyperglycemia, and increased liver glycogen production [3]. If this pathological progression is not treated immediately, the resultant muscle degeneration and impaired immune response can delay recovery and cause increased mortality [4]. Therefore, it is particularly important to clarify the state of these metabolic disorders and to optimize clinical therapies for these patients. Notably, optimal amino acid nutrition remains an open and controversial question.

It is widely acknowledged that plasma concentrations of most amino acids are greatly altered in sepsis [5, 6]. Freund et al. revealed that certain amino acids could be used as markers of the severity and prognosis of the disease process [7]. Vente et al. concluded that amino acid profiles showed non-specific trends and were therefore poor indicators of disease severity [8]. Together, these conclusions showed that disorders of amino acid metabolism intensified as the disease severity increased. In the course of sepsis treatment, enteral and parenteral nutrition are often used to supply essential amino acids and trace elements. Appropriate nutritional support, such as amino acid supplementation, can improve the recovery and outcomes of patients with sepsis [9]. However, the amino acid metabolic process is complex, involving many molecules and numerous biochemical metabolic pathways. Thus, a considerable controversy exists regarding which amino acids or molecules are most important, which therapeutic approaches to use and when these approaches should be used [10]. Therefore, better knowledge of the dynamic changes in a series of amino acids is necessary not only to make more appropriate estimations of amino acid requirements but also to provide a basis for sepsis nutritional support in intensive care units (ICUs). The aTRAP test is a high-throughput mass spectrometry method that can be used to describe the time courses of all amino acid-related molecules during the different stages of sepsis. In the present study, we measured dynamic changes in 42 amino acids in patients with systemic inflammatory response syndrome (SIRS) and sepsis and in healthy control subjects to investigate their amino acid concentration profiles.

## Materials and Methods

### Study subjects

This study was approved by the Ethics Committee of the Chinese PLA General Hospital (PLAGHHN2013001) and registered with ClinicalTrial.gov (NCT01818830). The patients or their family members were fully informed of the study details and agreed to participate in the study. All of the subjects were selected from among inpatients who were hospitalized between March 2012 and May 2013 in the Surgical ICU, Respiratory ICU, and Emergency ICU of Chinese PLA General Hospital. Patient inclusion criteria were as follows: (1) To be diagnosed with SIRS, the patients had to meet two or more criteria of the surviving sepsis campaigns of 2008 and 2012 [11, 12]; to be diagnosed with sepsis, the patients had to exhibit the simultaneous presence of SIRS and show evidence of a bacterial etiology. Severe sepsis was defined as sepsis with sepsis-induced organ dysfunction/tissue hypoperfusion or persistently low blood pressure following the administration of intravenous fluids. (2) Because this study focused on dynamic changes in 42 amino acids, all of the patients involved had to remain in the ICU for ≥14 days, or they must have died within 14 days. (3) The patients had to be 18 years old or older. The exclusion criteria were as follows: (1) parenteral nutrition within 48 h after ICU admission; (2) a history of diabetes or other metabolic-related diseases; (3) a history of chronic liver disease; (4) neutropenia (≤500 neutrophils/mm^3^); (5) human immunodeficiency virus (HIV) infection; and (6) refusal to take part in this study by the patients or their relatives. Based on the severity of their conditions, the sepsis patients were subdivided into sepsis and severe sepsis groups. The sepsis patients were also divided into survivor and non-survivor groups, with 28-day survival used as the cutoff. For the healthy control outpatients, acute and past chronic diseases were excluded. Moreover, we ensured that the healthy control subjects had not been hospitalized or taken any drugs during the previous 12 months and that they displayed normal results in physical checkups and lab examinations. The normal controls had a gender ratio of 12 males to 6 females and an age range of 46 ± 16 years.

### Samples and clinical data collection

Blood samples for the determination of serum amino acids were collected by venipuncture within 24 h after ICU admission. Moreover, intravenous blood sample collection was repeated on the mornings of days 3, 5, 7, 10, and 14 after ICU admission. The blood was centrifuged at 3,000 rpm for 15 min. The supernatants were transferred to Eppendorf tubes and stored at -80°C prior to analysis. At the same time, patient details were recorded, including age, gender, chief complaints for admission, past medical history, follow-ups of 28-day survivals, Acute Physiology and Chronic Health Evaluation (APACHE) II scores, Sequential Organ Failure Assessment (SOFA) scores, indexes of basic clinical lab testing (which included indexes of infection, such as white blood cell counts and C-reactive protein and procalcitonin levels), liver function (alanine transaminase, aspartate aminotransferase, total bilirubin, and direct bilirubin levels), renal function (blood urea nitrogen and serum creatinine levels), mechanical ventilation, continuous renal replacement treatment (CRRT), etiological factors, and underlying diseases.

### Reagents and chemicals

AB Sciex (AB Sciex, Foster City, CA, USA) provided the unlabeled amino acid standards, control plasma, and the aTRAQ reagent kit for the analysis of amino acids in physiological fluids. The reagent kit contained all of the necessary reagents to label amino acids with the aTRAQ tag, including the aTRAQ Reagent 121, isopropanol, formic acid, heptafluorobutyric acid, borate-labeling buffer (containing 20 μM norvaline), 10% sulfosalicylic acid (containing 400 μM norleucine), and 1.2% hydroxylamine solution. The reagent kit also contained an internal standard solution, which consisted of 44 amino acids labeled with the 113 aTRAQ tag (the concentration of each amino acid was 5 μM except for L-cystine, which was present at 2.5 μM). For quality control purposes, norleucine and norvaline (non-physiological amino acids) were added to the sulfosalicylic acid and borate-labeling buffer, respectively, to assess the extraction and labeling efficiencies of the assay.

### Separation and detection

The amino acids were separated by liquid chromatography using a Shimadzu Prominence UFLC system (LC 20AD-XR) with an AB Sciex C18 column (5 μm, 4.6 mm × 150 mm). The detection and identification were performed using an AB Sciex QTRAP 5500 tandem mass spectrometer that was operated in multiple reaction monitoring (MRM) mode. The acquisition and processing of all of the data were conducted using Analyst 1.5.2 software (AB Sciex). Specifically, the chromatographic separation of amino acids was performed using the AB Sciex C18 column at a temperature of 50°C. A binary gradient of water (mobile phase A) and methanol (mobile phase B), both of which contained 0.1% formic acid and 0.01% heptafluorobutyric acid, were delivered at a rate of 0.8 mL/min according to the program shown in S1 Table. The run time between injections was 18 min, which allowed all aTRAQ-tagged amino acids to be fully separated.

### Sample preparation and labeling with aTRAQ reagents

The amino acid analysis was performed in triplicate in accordance with the manufacturer’s protocol. A total of 40 μL of each serum sample was pipetted into a 1.5 mL Eppendorf tube. Next, 10 μL 10% sulfosalicylic acid was added to precipitate the proteins from the samples. The samples were vortexed and centrifuged at 10,000 g for 2 min, and 10 μL of the supernatant was then transferred to a clean tube. A total of 40 μL of labeling buffer was added to a 10 μL aliquot of supernatant, and the samples were vortexed and centrifuged for 1 min at 10,000 g. Then, 10 μL of the supernatant was pipetted into a clean tube and mixed with 5 μL of 121 aTRAQ tag for labeling, and the tubes were vortexed and centrifuged. Next, the tubes were incubated at room temperature for more than 30 min. To quench the labeling reaction, 5 μL hydroxylamine was added to each tube. The samples were completely dried in a centrifugal vacuum concentrator for approximately 1 h. The internal standard solution (32 μL) was added to each tube, and the samples were centrifuged at 15,000 g for 2 min. Finally, the labeled sample/internal standard mixture was transferred to an autosampler vial with a low volume insert (Agilent Technologies, Wilmington, DE).

### Quantification of amino acids

The amino acids were quantified using Analyst 1.5.2 software (AB Sciex). The amino acid concentrations were determined by dividing the analyte peak area by the peak area of the corresponding internal standard and then multiplying by the concentration of the internal standard. The MultiQuant 2.0.2 software (AB Sciex) was utilized for the analysis of raw data. The mass transitions for the amino acids and their corresponding internal standards are shown in S1 Table. The concentrations of norvaline (Nva) and norleucine (Nle) were measured to evaluate the recovery rates of the amino acids. From the analysis of 185 samples, the concentrations of these two amino acids were 94.94 ± 9.22 and 101.31 ± 15.60 μmol/L, respectively. The recovery percentages of Nva and Nle were 92.44± 8.98% and 102.44±15.77%. In addition, it should be noted that the total concentrations of certain groups of amino acids were calculated by summing the amino acids based on amino acid classification.

### Statistical analysis

Statistical analyses were conducted using SPSS 16.0 software (SPSS, Chicago, IL, USA), and P<0.05 was considered significant. The results for continuous variables with normal distributions were reported as the means ± standard deviations (SD). Student’s t-test was used to compare the means between two groups. Analysis of variance (ANOVA) was used to compare the means among multiple groups. LSD and SNK tests were used for data correction. The results for continuous variables that were not normally distributed were provided as medians (25th-75th percentiles) and compared using non-parametric tests. The results for qualitative variables were expressed as percentages and compared between groups using the chi-squared test. A Pearson correlation study was conducted to analyze the relevance of the post-admission day serum amino acid densities to the APACHE II and SOFA scores. The areas under receiver operating characteristic (ROC) curves were used to evaluate the outcome prediction value of sulfur-containing amino acids (SAAs).

## Results

### General information

**Fig 1:**
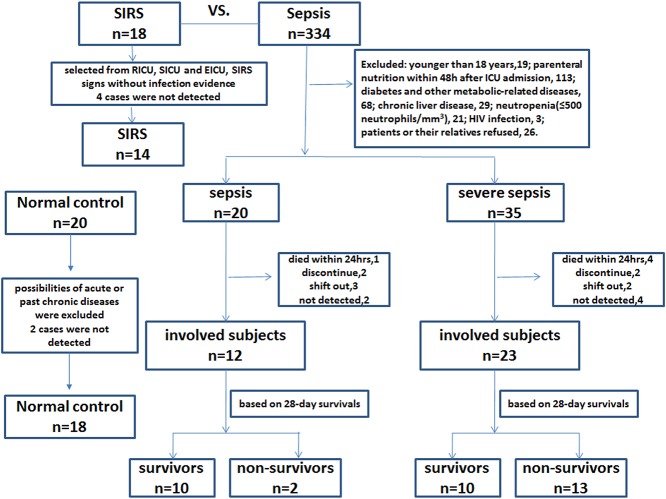
Trial profiles of the patients enrolled in our study. A total of 55 out of 334 septic patients were selected based on the relevant criteria. The sepsis group was further divided into sepsis (n = 12) and severe sepsis (n = 23) subgroups, as well as into survivor (n = 20) and non-survivor (n = 15) subgroups. Additionally, 18 normal controls and 14 SIRS were also included in this study.

A total of 14 patients with SIRS, 35 patients with sepsis, and 18 normal controls were selected, in accordance with the relevant criteria, to be formally included in this study (Fig. 1). The sepsis group was further divided into sepsis and severe sepsis subgroups, as well as into survivor and non-survivor subgroups. Table 1 provides a general summary of the subjects’ clinical characteristics. The serum procalcitonin (PCT) and blood urea nitrogen (BUN) levels and the APACHE II scores were higher in the sepsis and severe sepsis groups than those in the SIRS and sepsis groups, respectively (*P<0*.*05*). Compared with the sepsis group, the SOFA scores in the severe sepsis group were significantly higher (*P = 0*.*001*). Regarding the 28-day survivals, the mortality rates for the severe sepsis and sepsis groups were higher than those of the sepsis and SIRS groups, respectively (*P<0*.*05*). Additionally, the non-survivor group had higher APACHE II and SOFA scores than the survivor group (*P<0*.*05*). The non-survivor group had higher percentages of mechanical ventilation and post-operative trauma (*P<0*.*05*). There were no statistically significant differences for any other parameters.

**Table 1:** The clinical characteristics at ICU admission of patients involved in this study.

Characteristics	SIRS	Sepsis	P value	Sepsis (non-severe)	Severe sepsis	P value	Survivor	Non-survivor	P value
	N = 14	N = 35		N = 12	N = 23		N = 20	N = 15	
Age (years)	47±13	57±22	0.057	52±21	59±23	0.381	54±23	61±21	0.296
Gender (n, %)									
Male	9(64.3)	25(71.4)	0.624	9(75)	16(69.9)	0.735	14(70)	11(73.3)	0.829
Female	5(35.7)	10(28.6)		3(25)	7(30.4)		6(30)	4(26.7)	
Temperature (°C)	37.4±0.8	37.5±0.9	0.613	37.4±0.9	37.6±0.9	0.378	37.5±0.9	37.6±1.0	0.693
WBC counts (×10^9/L)	14.11±5.99	13.94±6.81	0.936	12.11±4.25	14.89±7.74	0.258	13.95±6.90	13.92±6.93	0.991
Serum CRP (mg/dL)	11.66±7.35	10.87±8.73	0.787	9.84±7.62	11.41±9.37	0.621	9.11±8.67	13.22±8.53	0.172
Serum PCT (ng/mL)	0.77(0.25–1.16)	2.81(0.51–10.78)	0.026	0.49(0.29–1.82)	5.15(1.01–16.43)	0.018	1.16(0.39–5.77)	4.68(1.15–16.42)	0.277
AST (U/L)	40.50(27.65–49.65)	26.40(16.80–49.00)	0.331	33.95(19.95–46.33)	24.00(15.70–141.90)	0.897	31.05(15.60–45.33)	26.4(17.5–254.70)	0.577
ALT (U/L)	35.20(13.45–83.40)	24.40(9.60–74.95)	0.536	29.85±27.34	87.79±138.06	0.204	42.91±41.78	103.10±170.13	0.253
TB (mmol/L)	16.69±7.11	21.51±17.86	0.339	23.36±162.30	20.48±190.81	0.691	19.49±13.11	24.64±23.85	0.467
DB (mmol/L)	7.67±4.26	12.36±11.92	0.168	12.80±110.08	12.11±128.10	0.9	11.09±9.23	14.59±16.10	0.522
BUN (mmol/L)	6.07±4.09	12.16±9.65	0.004	7.19±5.34	14.33±103.69	0.049	10.53±8.08	14.12±11.22	0.294
Serum creatinine (mmol/dL)	64.95(38.90–76.10)	74.05(49.10–112.50)	0.163	78.16±60.90	119.62±104.38	0.233	97.87±85.49	116.77±105.24	0.567
APACHE II score	9±3	18±8	<0.001	11±3	21±8	<0.001	14±7	22±8	0.007
SOFA score	-	8±4	-	5±3	10±4	0.001	7±4	10±5	0.017
MV (n, %)	7(50)	24(68.6)	0.223	7(58.3)	17(73.9)	0.346	11(55)	13(86.7)	0.046
CRRT (n, %)	2(14.3)	11(31.4)	0.219	2(16.7)	9(39.1)	0.174	6(25)	6(40)	0.344
Site of infection (n, %)									
Pulmonary	-	30(85.7)	-	11(91.7)	19(82.6)	0.467	17(85)	13(86.7)	0.889
Abdominal	-	10(28.6)	-	4(33.3)	6(26.1)	0.652	7(35)	3(20)	0.331
Urinary	-	9(25.7)	-	5(41.7)	4(17.4)	0.119	8(40)	1(6.7)	0.026
Trauma/postoperative	-	6(17.1)	-	2(16.7)	4(17.4)	0.957	0(0)	3(20)	0.036
Bacteremia	-	3(9)	-	0(0)	3(13)	0.191	2(33.3)	4(26.7)	0.195
Others	-	3(9)	-	0(0)	3(13)	0.191	0(0)	1(6.7)	0.241
Possible type of infection (n, %)									
Gram-positive bacteria	-	12(34.3)	-	3(25)	9(39.1)	0.403	7(35)	5(33.3)	0.918
Gram-negative bacteria	-	31(88.6)	-	12(100)	19(82.6)	0.125	18(90)	13(86.7)	0.759
Fungi	-	19(54.3)	-	5(41.7)	14(60.9)	0.279	10(50)	9(60)	0.557
Predisposing factors (n, %)									
Hypertension	1(7.1)	12(34.3)	0.052	5(41.7)	7(30.4)	0.506	6(30)	6(40)	0.537
COPD	0(0)	3(8.6)	0.258	1(8.3)	2(8.7)	0.971	2(10)	1(6.7)	0.727
Coronary heart disease	1(7.1)	9(25.7)	0.145	3(25)	6(25.1)	0.944	6(30)	3(20)	0.503
Immunosuppressed condition	0(0)	5(14.3)	0.136	0(0)	5(21.7)	0.081	1(5)	4(26.7)	0.07
Nervous system disease	0(0)	2(5.7)	0.361	0(0)	2(8.7)	0.293	1(5)	1(6.7)	0.833
CKD	0(0)	4(11.4)	0.187	1(8.3)	3(13)	0.678	3(15)	1(6.7)	0.443
28-day mortality rate (n, %)	1(7.1)	15(42.9)	<0.001	2(16.7)	13(56.5)	0.024	-	-	-

Normally distributed quantitative data are presented as the means ± SD. Non-normally distributed quantitative data are presented as the medians (25th-75th percentiles). Qualitative data are presented as n (%).

### The differences in amino acid profiles with SIRS or sepsis on ICU admission

The amino acid profiles significantly changed with SIRS and sepsis (Table 2). Compared with the normal controls, certain amino acid levels tended to decrease, but others increased or were stable. The serum concentrations of some amino acids (carnosine, citrulline, histidine, ornithine, proline, sarcosine, threonine, tryptophan, tyrosine, valine, isoleucine, cystathionine, lysine, anserine, phosphoethanolamine, asparagine, and leucine) were significantly lower in SIRS and sepsis patients than in the normal controls (*P<0*.*05*). In contrast, the concentrations of other amino acids (arginine, glutamine, phenylalanine, taurine, aspartic acid, ethanolamine, homocitrulline, and glutamic acid) were significantly higher in SIRS and sepsis patients than in the normal controls (*P<0*.*05*). Compared with the normal control and sepsis groups, the δ-hydroxylysine and phospho-L-serine levels were significantly higher and the hydroxyproline levels were lower in the SIRS group (*P<0*.*05*). In addition, the levels of essential amino acids, branched-chain amino acids (BCAAs), SAAs, and branched-chain/aromatic amino acids in the SIRS and sepsis groups were significantly higher than those of the normal control group (*P<0*.*05*).

**Table 2:** Comparison of amino acid serum concentrations among the normal control, SIRS, and sepsis groups on the day of ICU admission.

Amino acids	Normal control	SIRS	Sepsis	P value
μmol/L	N = 18	N = 14	N = 35	
Increasing tendency				
Arginine	26.88±14.08* ^	77.79±16.79^	67.86±27.96*	<0.001
Aspartic acid	6.73±1.39* ^	41.17±22.70^	21.30±4.10*	<0.001
Ethanolamine	6.89±1.60*	7.97±2.64#	10.92±4.12* #	<0.001
Glutamine	165.62±228.05* ^	480.41±132.69^	428.33±169.32*	<0.001
Glutamic acid	72.55±19.72*	115.25±60.75	138.77±98.17*	0.018
Homocitrulline	0.35±0.21*	0.44±0.94#	1.30±1.77* #	0.033
Phenylalanine	67.53±9.99* ^	110.32±30.40^	116.39±44.70*	<0.001
Taurine	61.90±11.03* ^	101.40±48.98^	90.51±55.50*	0.038
EAAs	1105.71±200.70* ^	918.77±289.44^	841.98±238.72*	0.003
BCAAs	601.96±119.34* ^	456.44±168.66^	417.51±134.85*	<0.001
SAAs	101.97±16.23* ^	148.15±53.60^	147.72±63.52*	0.01
BCAA/AAA ratio	3.13±0.47* ^	2.17±0.45^	2.07±0.59*	<0.001
Decreasing trendency				
Anserine	0.22±0.07*	0.23±0.09#	0.15±0.05* #	0.001
Asparagine	67.18±12.89*	61.06±20.02	50.88±18.66*	0.011
Carnosine	0.12±0.005* ^	0.07±0.04^	0.08±0.03*	<0.001
Citrulline	34.01±9.76* ^	11.95±3.62^	14.54±7.01*	<0.001
Cystathionine	4.32±1.38* ^	2.15±0.65# ^	3.49±1.42* #	<0.001
Histidine	85.31±12.08* ^	63.53±15.10^	63.31±27.58*	0.002
Isoleucine	102.73±26.41* ^	77.88±37.50^	67.88±23.38*	0.002
Leucine	166.87±35.56*	144.24±61.98	116.80±40.64*	0.003
Lysine	195.98±38.57*	179.96±67.76#	143.46±48.25* #	0.004
Ornithine	134.13±42.57* ^	74.00±38.20^	78.16±29.17*	<0.001
Phosphoethanolamine	9.31±2.17*	10.28±2.46#	1.18±1.40* #	<0.001
Proline	182.55±51.75* ^	128.94±49.64^	143.97±66.34*	0.028
Sarcosine	18.23±7.37* ^	1.48±1.14^	2.46±3.31*	<0.001
Threonine	160.07±49.47*^	112.12±29.35^	107.82±45.84*	0.001
Tryptophan	59.25±10.85* ^	40.22±12.06^	38.81±17.20*	<0.001
Tyrosine	66.88±19.96* ^	54.65±11.87^	53.07±17.31*	0.029
Valine	332.36±61.73* ^	234.33±75.09^	232.83±72.49*	<0.001
SIRS-specific expression				
δ-Hydroxylysine	1.38±0.70^	2.53±0.44^ #	1.51±0.43#	<0.001
Hydroxy-L-proline	25.43±12.71^	12.24±7.59^	20.52±16.76	0.034
Phosphoserine	49.12±121.78^	378.18±321.45^ #	1.82±1.98#	<0.001
Stable trendency				
3-Methyl-L-histidine	3.69±1.62	4.26±3.02	5.47±5.11	0.303
α-Aminoadipic acid	1.39±0.45	1.46±0.54	1.56±0.65	0.615
Argininosuccinic acid	0.01±0.06	0.14±0.11	0.12±0.08	0.376
β-Aminoisobutyric acid	2.68±1.54	2.21±1.59	7.07±11.27	0.085
Cystine	18.97±6.17	26.83±11.48	21.54±10.70	0.08
Glycine	274.60±63.23	261.43±179.92	306.59±173.35	0.616
NEAAs	1520.75±325.33	1818.24±482.42	1710.66±661.94	0.282
GAAs	1807.16±355.30	2017.63±529.65	1901.08±710.35	0.599
AAAs	193.65±37.29	205.18±43.26	208.27±12.87	0.661
1-Methyl-L-histidine	2.92±1.57	2.36±0.93	2.36±1.30	0.327
α-Amino-n-butyric acid	22.31±7.19	19.96±10.53	16.00±11.07	0.116
Alanine	400.74±108.33	375.10±169.13	333.25±164.41	0.399
Methionine	20.93±7.30	19.71±10.34	17.99±10.33	0.595
Serine	152.74±36.32	131.78±44.42	126.35±47.63	0.142
β-Alanine	18.45±3.93	16.05±2.60	18.88±5.00	0.125
γ-Amino-n-butyric acid	0.28±0.25	0.41±0.31	0.30±0.23	0.323
Homocysteine	0.18±0.16	0.22±0.13	0.16±0.17	0.517

AAs, amino acids; EAAs, essential AAs; NEAAs, nonessential AAs; GAAs, glycogenic AAs; BCAAs, branched-chain AAs; AAAs, aromatic AAs; SAAs, sulfur-containing AAs.

* Indicates sepsis vs. normal control groups, P<0.05;

# indicates sepsis vs. SIRS groups, P<0.05;

^ indicates SIRS vs. normal control groups, P<0.05.

### Comparison of the amino acid densities between the sepsis and severe sepsis groups

**Fig 2:**
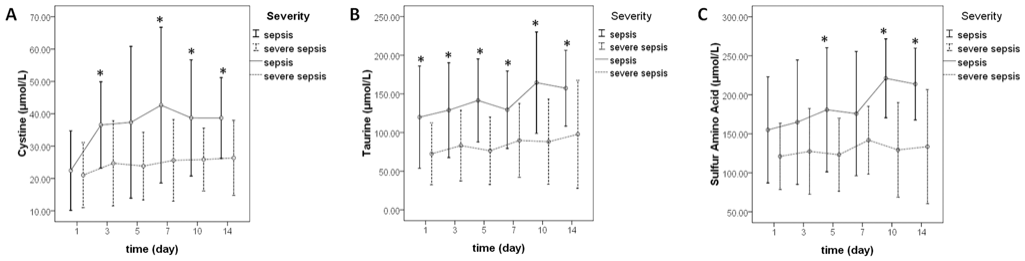
The dynamic changes in cystine (A), taurine (B), and sulfur-containing amino acid (C) levels between the sepsis and severe sepsis groups on days 1, 3, 5, 7, 10, and 14. *P<0.05.

As sepsis progressed and the severity gradually worsened, the concentration levels of various amino acids increased, decreased, or fluctuated (S3 Table). Severe sepsis resulted in higher concentrations of some amino acids compared with sepsis (3-methyl-L-histidine, α-aminoadipic acid, α-amino-n-butyric acid, argininosuccinic acid, β-amino-isobutyric acid, carnosine, cystathionine, glutamine, phenylalanine, and proline) at certain time points (*P<0*.*05*). In contrast, the concentration levels of arginine, asparagine, aspartic acid, cystine, glutamic acid, leucine, serine, taurine, tryptophan, BCAAs, and SAAs, as well as the BCAA/AAA ratio (the ratio of BCAAs to AAAs), were significantly lower at certain time points during the occurrence of severe sepsis (*P<0*.*05*). Specifically, the serum concentrations of taurine in the severe sepsis group were significantly lower than those in the sepsis group at all 6 time points (Fig. 2B). Moreover, the concentrations of cystine and SAAs in the severe sepsis group were significantly lower than those in the sepsis group at 4 time points and 3 time points, respectively (Fig. 2A and C).

### Comparison of the amino acid densities between the survivor and non-survivor groups

**Fig 3:**
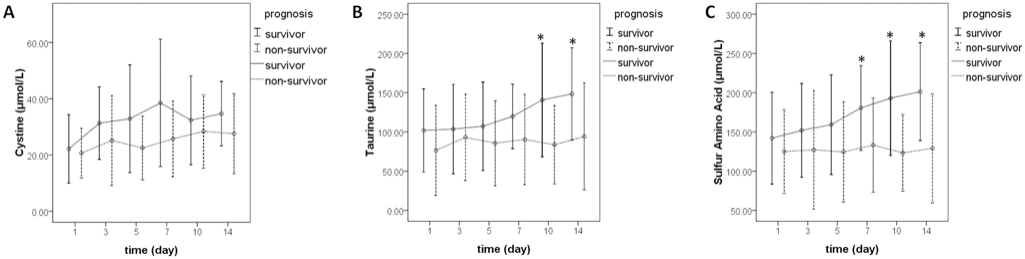
The dynamic changes in cystine (A), taurine (B), and sulfur-containing amino acid (C) levels between survivors and non-survivors on days 1, 3, 5, 7, 10, and 14. *P<0.05.

To explore the metabolic dysfunction of patients with poor prognoses, we compared the serum concentrations of amino acids between survivors and non-survivors based on a 28-day survival (S4 Table). The serum concentrations of α-aminoadipic acid, cystathionine, ethanolamine, and phenylalanine in non-survivors at certain time points were higher and statistically significant (*P<0*.*05*). Compared with survivors, serum concentrations of arginine, glutamic acid, serine, taurine, and tryptophan, as well as the BCAA/AAA ratio, in non-survivors were significantly lower (*P<0*.*05*) at certain time points. Fig. 3 shows the trends in cystine (A), taurine (B), and SAA (C) serum concentrations.

### Sulfur-containing amino acids and relevant scoring systems for patients with sepsis

**Fig 4:**
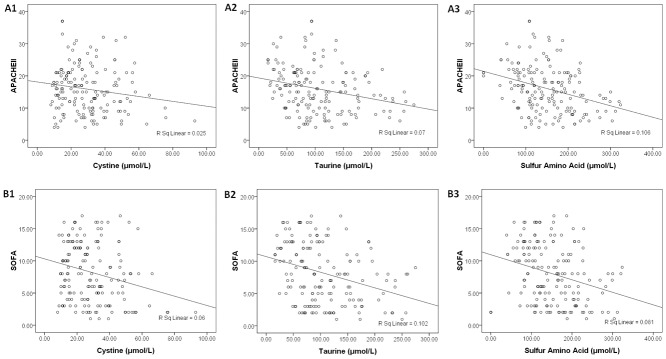
The relationships between the APACHE II (A) and SOFA (B) scoring systems and the serum concentrations of cystine (1), taurine (2), and sulfur-containing amino acids (3) at all time points post-admission.

APACHE II and SOFA scores were assigned to all of the post-admission time points of sepsis patients. The serum levels of cystine, taurine, and SAAs decreased as these scores increased. The Pearson correlation analysis showed a weak negative correlation between the scores and the levels of SAAs. The correlation coefficients between the APACHE II scores and cystine, taurine, and SAA levels were -0.157 (*P = 0*.*04*), -0.265 (*P<0*.*001*), and -0.325 (*P<0*.*001*), respectively (Fig. 4A). The correlation coefficients between the SOFA scores and cystine, taurine, and SAA levels were -0.244 (*P = 0*.*001*), -0.319 (*P<0*.*001*), and -0.285 (*P<0*.*001*), respectively (Fig. 4B).

### Outcome prediction value of sulfur-containing amino acids

**Fig 5:**
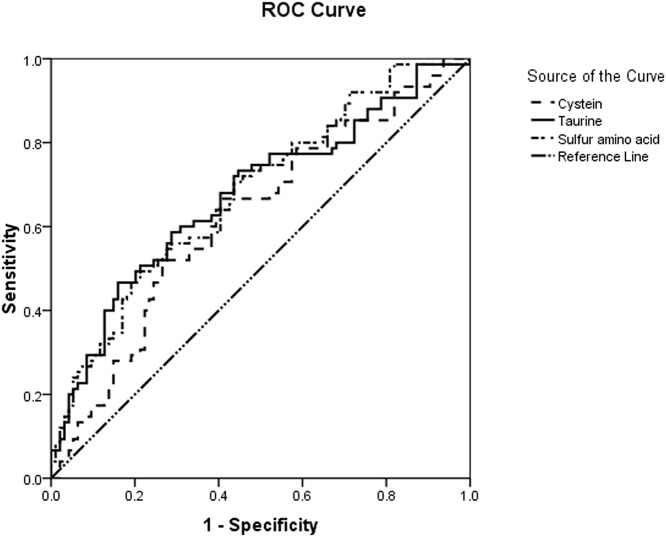
ROC curves for the outcome prediction value of the cystine, taurine, and sulfur-containing amino acid levels and the APACHE II and SOFA scoring systems at all time points post-admission. Sensitivity means true positive rate; 1-specificity means false positive rate; the 45-degree reference line stands for the chance diagonal, which means a diagnostic test is completely free of diagnostic value (the area under the curve equals 0.5).

Fig. 5 displays the ROC curve values for the outcome prediction value of SAAs. The areas under the curves (AUCs) for the detected indicators that showed statistically significant differences between the survivor and non-survivor groups are displayed in Table 3. The areas under the ROC curves of cysteine, taurine, and SAA levels denoting prognosis measured 0.623, 0.674, and 0.678, respectively. The ROCs of the SOFA and APACHE II scores were larger than the former three parameters.

**Table 3:** Areas under the ROC curves as the outcome prediction values of sulfur-containing amino acids.

Variable	AUC	Std. Error	P value	Asymptotic 95% Confidence Interval		Cutoff point	Sen	Spe	PPV	NPV	YI
				Lower limit	Upper limit						
Cystine	0.623	0.043	0.006	0.538	0.708	26.61	0.667	0.596	0.553	0.599	0.263
Taurine	0.674	0.042	<0.001	0.591	0.757	68.38	0.467	0.84	0.686	0.533	0.307
Sulfur-containing amino acids	0.678	0.041	<0.001	0.598	0.759	112.91	0.467	0.809	0.647	0.528	0.276
APACHE II	0.857	0.026	<0.001	0.806	0.907	15.5	0.833	0.746	0.711	0.773	0.579
SOFA	0.86	0.027	<0.001	0.807	0.914	8.5	0.8	0.833	0.782	0.752	0.633

Sen, sensitivity; Spe, specificity; PPV, positive predictive value; NPV, negative predictive value; YI, Youden’s index.

The “cutoff point” is defined as the maximum point of Youden’s index.

## Discussion

Using high-throughput mass spectrometry, our study first showed that the dynamic concentration profiles of amino acids in patients with sepsis exhibit significant changes between SIRS and sepsis patients during disease exacerbation or with poor prognosis. However, the amino acid concentration profiles are not consistent for any stage of sepsis progression. This result fully revealed the complexity of amino acid metabolism during sepsis. Specifically, disorders affecting SAA metabolism may have greater pathophysiological significance.

The catabolic rates of patients with sepsis are significantly higher than their anabolic rates; this difference results from neuroendocrine responses and is closely related to the activities of cytokines and some inflammatory mediators [4]. The severity of sepsis and the individual responses lead to inconsistent severities of metabolic differences. Disrupted metabolic balance and inappropriate nutrition therapy lead to the phenomenon of "auto-cannibalism" [13]. Amino acids are protein metabolites and are also synthetic precursors of proteins. Theoretically, the metabolic spectrum of amino acids changes when sepsis occurs. This study revealed that the concentrations of carnosine, citrulline, histidine, ornithine, proline, sarcosine, threonine, tryptophan, tyrosine, valine, isoleucine, cystathionine, lysine, anserine, phosphoethanolamine, asparagine, leucine, BCAAs, and SAAs, as well as the BCAA/AAA ratio, were lower in the SIRS and sepsis groups, but the serum levels of arginine, glutamine, phenylalanine, taurine, aspartic acid, ethanolamine, homocitrulline, and glutamic acid were higher in these groups. Carnosine and anserine have been shown to have antioxidant effects [14]. The lower concentrations of these amino acids results in increased levels of oxidative stress. Citrulline and threonine are relevant to gastrointestinal disorders during sepsis [15, 16]. Alexander et al. [17] showed that asparagine levels are significantly associated with better neutrophil function. The lower levels of BCAAs, namely leucine, isoleucine, and valine, have been shown to promote protein catabolism and to reduce muscle protein synthesis [18]. During sepsis, the consumption of proline, histidine, and ornithine exceeds protein synthesis, but arginine, glutamine, and glutamic acid levels remain high. This balance plays an important role in the regulation of acid/base homeostasis, fibroblast, lymphocyte, and enterocyte growth, and glutamine concentration preservation in skeletal muscle [19].

SAA levels are well known to change during sepsis [20, 21]. From our results, we demonstrated that severe sepsis patients and non-survivors had lower SAA concentrations. SAAs include cysteine, cystine, taurine, and methionine. Animal experiments have confirmed that N-acetylcysteine can reduce oxidative stress damage and the release of inflammatory mediators, which is beneficial to sepsis recovery [22, 23]. Other experimental studies have shown that acetylcysteine can reduce organ injury [24] and improve survival [25]. The role of cysteine in the treatment of sepsis has also been confirmed in clinical trials [26, 27]. Cysteine and cystine can be interconverted in the body. The detection kit used in this study only detected the serum levels of cystine. The severe sepsis group had lower concentrations of cystine than the sepsis group; this difference was statistically significant on days 3, 7, 10, and 14. Although the non-survivors also had lower serum concentrations, the difference was not statistically significant at any time point. The changes in cystine concentrations may have indirectly reflected changes in the cysteine levels.

Many studies have shown that the concentrations of taurine decrease when sepsis occurs [28, 29]. In this study, we obtained a similar result, in which the serum taurine levels in the severe sepsis group were significantly lower than those in the sepsis group at various time points. Additionally, serum taurine levels in the non-survivor group were lower than those in the survivor group. Several animal experiments have confirmed that anti-oxidative damage is the main role of taurine [30, 31]. The oxidation reaction consumes large quantities of taurine, which leads to lower taurine levels. Ekremoglu et al. also observed that taurine has considerable anti-inflammatory effects [31], thereby suggesting that a severe inflammatory response also contributes to taurine consumption. Therefore, it may be useful to supplement SAAs during sepsis.

From the discussion above, we know that the SAAs cysteine and taurine decrease with increasing sepsis severity. Beale et al. [32] showed that sepsis patients receiving an experimental supplement have a significantly faster decline in SOFA scores over time compared with controls, thereby suggesting that some relationship may exist between the levels of SAAs and the scoring system. Our analysis revealed a weak correlation between the concentrations of SAAs and the APACHE II and SOFA scores. This result suggested that SAAs are beneficial in the patient's nutritional therapy and that patients with higher scores may have low levels of SAAs in the body. However, other amino acids did not exhibit any correlation with the SOFA or APACHE II scores. Furthermore, ROC analysis demonstrated that the changes in SAAs levels were statistically relevant to outcome prediction. However, the concentrations of cystine, taurine and SAAs were not as accurate as SOFA and APACHE II scores for the assessment of septic prognosis.

The present study possessed certain limitations. (1) Although continuous monitoring occurred over multiple time points, the sample size of this study was limited, including only 35 sepsis patients. In accordance with the different prognoses, we found that the SAA concentrations in the non-survivor group were lower than those in the survivor group at all time points but that these differences were not statistically significant at certain time points. One explanation for the lack of statistical significance could be the heterogeneity among the sepsis patients, which could have led to many different metabolic disorders. In addition, the limited number of patients also resulted in the large error bars in the figures. It would be necessary to use a larger sample size to validate our conclusions. (2) According to the relationship between cystine and cysteine and in conjunction with the literature, cysteine may possess more pathophysiological significance during the course of sepsis than cystine. However, this study examined the concentration of cystine due to the detection limit of the aTRAQ kit. If conditions permit, the concentration of cysteine should be monitored. (3) Renal impairment has been reported to increase taurine levels [8, 33], whereas CRRT is well known to remove amino acids [34]. In this study, we did not exclude the influence of CRRT on amino acid metabolism, especially on that of taurine. If possible, further analysis should be conducted in the future to determine how CRRT affects the study results.

## Conclusion

The amino acid metabolic profiles clearly changed in sepsis patients. The SAA concentrations, especially of taurine, decreased as the sepsis severity increased, and the concentrations of these amino acids were significantly lower in the non-survivor group than in the survivor group. This study provides a theoretical basis for the nutritional support of sepsis treatments. Supplemental treatments that include SAAs may be useful for recovery, but clinical RCTs are needed to confirm these conclusions.

## Supporting Information

S1 Table LC gradient for the assay. (DOC)

S2 Table Mass transitions for amino acids and their corresponding internal standards. (DOC)

S3 Table Comparison of amino acid serum concentrations between sepsis (12 cases) and severe sepsis (23 cases) groups. (DOC)

S4 Table Comparison of amino acid serum concentrations between survivor (20 cases) and non-survivor (15 cases) groups. (DOC)
