# Supplementary material for: Dynamic Changes in Amino Acid Concentration Profiles in Patients with Sepsis
Source: PLoS One. 2015 Apr 7;10(4):e0121933. doi: 10.1371/journal.pone.0121933 (PMC4388841; doi:10.1371/journal.pone.0121933)
Supplement: S1 Table — (DOC) [file pone.0121933.s001.doc]

S1 Table LC gradient for the assay.

| **Total Time** | **%Mobile phase A** | **%Mobile phase B** |
| --- | --- | --- |
| 0.0 | 98 | 2 |
| 6.0 | 60 | 40 |
| 10.0 | 60 | 40 |
| 11.0 | 10 | 90 |
| 12.0 | 10 | 90 |
| 13.0 | 98 | 2 |
| 18.0 | 98 | 2 |
