# Supplementary material for: Dynamic Changes in Amino Acid Concentration Profiles in Patients with Sepsis
Source: PLoS One. 2015 Apr 7;10(4):e0121933. doi: 10.1371/journal.pone.0121933 (PMC4388841; doi:10.1371/journal.pone.0121933)
Supplement: S2 Table — (DOC) [file pone.0121933.s002.doc]

S2 Table Mass transitions for amino acids and their corresponding internal standards.

| **Amino acid** | **Analytes** | | |  | **Internal standards** | | |
| --- | --- | --- | --- | --- | --- | --- | --- |
|  | Abbreviation | Q1 mass | Q3 mass |  | Abbreviation | Q1 mass | Q3 mass |
| O-phospho-L-serine | PSer | 334.1 | 121.1 |  | PSer_IS | 326.1 | 113.1 |
| O-phosphoethanolamine | PEtN | 290.1 | 121.1 |  | PEtN_IS | 282.1 | 113.1 |
| Taurine | Tau | 274.1 | 121.1 |  | Tau_IS | 266.1 | 113.1 |
| L-asparagine | Asn | 281.2 | 121.1 |  | Asn_IS | 273.2 | 113.1 |
| L-serine | Ser | 254.2 | 121.1 |  | Ser_IS | 246.2 | 113.1 |
| Glycine | Gly | 224.1 | 121.1 |  | Gly_IS | 216.1 | 113.1 |
| Hydroxy-L-proline | Hyp | 280.1 | 121.1 |  | Hyp_IS | 272.1 | 113.1 |
| Ethanolamine | EtN | 210.2 | 121.1 |  | EtN_IS | 202.2 | 113.1 |
| L-glutamine | Gln | 295.2 | 121.1 |  | Gln_IS | 287.2 | 113.1 |
| L-aspartic acid | Asp | 282.2 | 121.1 |  | Asp_IS | 274.2 | 113.1 |
| L-citrulline | Cit | 324.2 | 121.1 |  | Cit_IS | 316.2 | 113.1 |
| L-threonine | Thr | 268.2 | 121.1 |  | Thr_IS | 260.2 | 113.1 |
| sarcosine | Sar | 238.2 | 121.1 |  | Sar_IS | 230.2 | 113.1 |
| β-alanine | bAla | 238.2 | 121.1 |  | bAla_IS | 230.2 | 113.1 |
| L-alanine | Ala | 238.2 | 121.1 |  | Ala_IS | 230.2 | 113.1 |
| L-glutamic acid | Glu | 296.2 | 121.1 |  | Glu_IS | 288.2 | 113.1 |
| L-histidine | His | 304.2 | 121.1 |  | His_IS | 296.2 | 113.1 |
| 1-methyl-  L-histidine | 1MHis | 318.2 | 121.1 |  | 1MHis_IS | 310.2 | 113.1 |
| 3-methyl-  L-histidine | 3MHis | 318.2 | 121.1 |  | 3MHis_IS | 310.2 | 113.1 |
| argininosuccinic acid | Asa | 439.2 | 121.1 |  | Asa_IS | 431.2 | 113.1 |
| homocitrulline | Hcit | 338.2 | 121.1 |  | Hcit_IS | 330.2 | 113.1 |
| L-anserine | Ans | 389.2 | 121.1 |  | Ans_IS | 381.2 | 113.1 |
| L-carnosine | Car | 375.2 | 121.1 |  | Car_IS | 367.2 | 113.1 |
| L-α-aminoadipic acid | Aad | 310.2 | 121.1 |  | Aad_IS | 302.2 | 113.1 |
| γ-amino-n-butyric acid | GABA | 252.2 | 121.1 |  | GABA_IS | 244.2 | 113.1 |
| D,L-β-amino-isobutyric acid | bAib | 252.2 | 121.1 |  | bAib_IS | 244.2 | 113.1 |
| L-α-amino-n-butyric acid | Abu | 252.2 | 121.1 |  | Abu_IS | 244.2 | 113.1 |
| L-arginine | Arg | 323.2 | 121.1 |  | Arg_IS | 315.2 | 113.1 |
| L-proline | Pro | 264.2 | 121.1 |  | Pro_IS | 256.2 | 113.1 |
| L-ornithine | Orn | 429.2 | 121.1 |  | Orn_IS | 413.2 | 113.1 |
| cystathionine | Cth | 519.2 | 121.1 |  | Cth_IS | 503.2 | 113.1 |
| L-cystine | Cys | 537.2 | 121.1 |  | Cys_IS | 521.2 | 113.1 |
| δ-hydroxylysine | Hyl | 459.3 | 121.1 |  | Hyl_IS | 443.3 | 113.1 |
| L-lysine | Lys | 443.3 | 121.1 |  | Lys_IS | 427.3 | 113.1 |
| L-methionine | Met | 298.1 | 121.1 |  | Met_IS | 290.1 | 113.1 |
| L-valine | Val | 266.2 | 121.1 |  | Val_IS | 258.2 | 113.1 |
| L-norvaline | Nva | 266.2 | 121.1 |  | Nva_IS | 258.2 | 113.1 |
| L-tyrosine | Tyr | 330.2 | 121.1 |  | Tyr_IS | 322.2 | 113.1 |
| L-homocystine | Hcy | 565.2 | 121.1 |  | Hcy_IS | 549.2 | 113.1 |
| L-isoleucine | Ile | 280.2 | 121.1 |  | Ile_IS | 272.2 | 113.1 |
| L-leucine | Leu | 280.2 | 121.1 |  | Leu_IS | 272.2 | 113.1 |
| L-norleucine | Nle | 280.2 | 121.1 |  | Nle_IS | 272.2 | 113.1 |
| L-phenylalanine | Phe | 314.2 | 121.1 |  | Phe_IS | 306.2 | 113.1 |
| L-tryptophan | Trp | 353.2 | 121.1 |  | Trp_IS | 345.2 | 113.1 |
