# Supplementary material for: Dynamic Changes in Amino Acid Concentration Profiles in Patients with Sepsis
Source: PLoS One. 2015 Apr 7;10(4):e0121933. doi: 10.1371/journal.pone.0121933 (PMC4388841; doi:10.1371/journal.pone.0121933)
Supplement: S3 Table — (DOC) [file pone.0121933.s003.doc]

S3 Table Comparison of amino acid serum concentrations between sepsis (12 cases) and severe sepsis (23 cases) groups.

| **Amino acid**  μmol/L | **Day 1** | **Day 3** | **Day 5** | **Day 7** | **Day 10** | **Day 14** |
| --- | --- | --- | --- | --- | --- | --- |
| 1-Methyl-L-Histidine |  |  |  |  |  |  |
| sepsis | 2.39±1.32 | 3.51±2.43 | 2.90±1.03 | 2.56±0.92 | 3.15±1.68 | 3.76±2.48 |
| severe sepsis | 2.33±1.34 | 2.09±0.72 | 2.20±1.03 | 2.25±1.11 | 2.12±1.20 | 2.51±1.30 |
| 3-Methyl-L-Histidine |  |  |  |  |  |  |
| sepsis | 4.16±4.56 | 3.98±3.34* | 3.90±2.95* | 4.44±4.22 | 4.74±3.76 | 3.90±2.30* |
| severe sepsis | 6.43±5.43 | 7.56±5.49* | 8.14±6.60* | 7.35±5.48 | 7.13±6.51 | 7.67±6.01* |
| α-aminoadipic acid |  |  |  |  |  |  |
| sepsis | 1.27±0.45* | 1.30±0.60* | 1.51±0.77 | 1.65±0.60 | 1.33±0.52 | 1.53±0.73 |
| severe sepsis | 1.78±0.70* | 2.19±1.32* | 2.26±1.84 | 2.52±2.02 | 2.13±1.56 | 2.07±1.59 |
| α-amino-n-butyric acid |  |  |  |  |  |  |
| sepsis | 11.11±6.2* | 13.97±6.91 | 12.83±5.90 | 18.47±17.79 | 17.88±12.09 | 16.04±8.53 |
| severe sepsis | 19.83±12.67* | 18.84±13.38 | 18.14±15.53 | 22.55±23.82 | 18.31±21.69 | 19.30±21.95 |
| Alanine |  |  |  |  |  |  |
| sepsis | 281.34±79.79 | 299.99±46.64 | 386.10±142.15 | 328.22±129.38 | 351.27±93.14 | 344.99±88.43 |
| severe sepsis | 371.32±239.60 | 324.68±127.52 | 318.40±111.81 | 343.84±130.09 | 317.98±110.36 | 347.89±131.56 |
| Anserine |  |  |  |  |  |  |
| sepsis | 0.15±0.06 | 0.19±0.06 | 0.22±0.07* | 0.20±0.08 | 0.16±0.07 | 0.18±0.06 |
| severe sepsis | 0.15±0.04 | 0.16±0.05 | 0.14±0.06* | 0.16±0.06 | 0.16±0.06 | 0.19±0.07 |
| Arginine |  |  |  |  |  |  |
| sepsis | 72.87±27.91 | 85.34±26.61 | 84.98±31.49 | 108.71±18.71 | 99.51±42.11* | 85.31±28.06 |
| severe sepsis | 64.19±28.37 | 67.12±27.07 | 75.56±35.02 | 20.98±37.70 | 70.55±22.85* | 68.84±19.08 |
| Argininosuccinic acid |  |  |  |  |  |  |
| sepsis | 0.06±0.04* | 0.09±0.09* | 0.069±0.07 | 0.10±0.07 | 0.10±0.05* | 0.09±0.05* |
| severe sepsis | 0.16±0.08* | 0.18±0.14* | 0.15±0.14 | 0.15±0.11 | 0.15±0.10* | 0.15±0.11* |
| Asparagine |  |  |  |  |  |  |
| sepsis | 47.33±15.17 | 57.64±19.40 | 61.04±22.74 | 70.76±40.25 | 70.78±15.94* | 66.41±20.69 |
| severe sepsis | 53.47±20.98 | 54.41±20.48 | 61.32±24.27 | 64.94±23.69 | 54.37±18.57* | 56.48±18.42 |
| Aspartic acid |  |  |  |  |  |  |
| sepsis | 47.05±18.75 | 46.11±14.38 | 51.34±25.75 | 38.98±12.81 | 48.31±22.34* | 48.20±22.38 |
| severe sepsis | 33.22±21.76 | 35.29±20.90 | 35.45±27.42 | 33.14±22.22 | 31.32±20.69* | 33.02±18.63 |
| β-aminoisobutyric acid |  |  |  |  |  |  |
| sepsis | 3.12±5.20* | 2.50±4.77 | 2.58±4.83* | 2.69±4.82 | 3.75±7.44 | 3.33±4.85 |
| severe sepsis | 9.96±13.65* | 10.51±18.01 | 15.33±31.67* | 10.97±25.03 | 6.59±14.82 | 7.54±15.10 |
| β-Alanine |  |  |  |  |  |  |
| sepsis | 17.68±4.53 | 18.08±6.07 | 19.19±6.94 | 17.35±4.62 | 16.86±4.62 | 17.52±3.63 |
| severe sepsis | 19.75±5.30 | 16.53±3.23 | 16.53±3.82 | 15.24±4.06 | 16.91±5.33 | 17.77±3.42 |
| carnosine |  |  |  |  |  |  |
| sepsis | 0.07±0.02 | 0.10±0.04 | 0.06±0.02* | 0.07±0.04 | 0.07±0.03 | 0.08±0.03 |
| severe sepsis | 0.08±0.03 | 0.10±0.07 | 0.09±0.03* | 0.10±0.05 | 0.10±0.03 | 0.09±0.04 |
| Citrulline |  |  |  |  |  |  |
| sepsis | 12.70±6.34 | 18.52±7.98 | 17.00±8.30 | 16.89±8.08 | 17.96±7.39 | 18.74±8.91 |
| severe sepsis | 15.90±7.37 | 16.54±7.63 | 17.76±9.63 | 17.50±8.13 | 18.22±11.18 | 16.20±7.17 |
| Cystathionine |  |  |  |  |  |  |
| sepsis | 3.00±0.76 | 3.15±0.92* | 3.56±1.30 | 3.85±1.25 | 3.71±1.19 | 3.43±0.59* |
| severe sepsis | 3.84±1.45 | 4.23±1.09* | 4.19±1.75 | 4.40±1.29 | 4.25±1.64 | 4.37±1.57* |
| Cystine |  |  |  |  |  |  |
| sepsis | 24.45±12.28 | 36.54±13.33* | 37.36±23.43 | 42.65±24.05* | 38.71±17.95* | 38.69±12.46* |
| severe sepsis | 21.04±10.06 | 25.25±14.23* | 27.18±18.94 | 26.20±13.78* | 26.41±11.11* | 26.91±12.76* |
| Ethanolamine |  |  |  |  |  |  |
| sepsis | 9.80±2.43 | 11.56±3.51 | 13.16±4.84 | 11.70±5.63 | 12.36±4.15 | 11.47±4.72 |
| severe sepsis | 11.75±4.93 | 13.46±6.31 | 12.27±6.63 | 13.12±4.35 | 13.05±4.97 | 13.38±4.61 |
| γ-Amino-n-Butyric Acid |  |  |  |  |  |  |
| sepsis | 0.23±0.12 | 0.31±0.20 | 0.26±0.15 | 0.30±0.15 | 0.27±0.24 | 0.35±0.29 |
| severe sepsis | 0.36±0.28 | 0.32±0.17 | 0.28±0.19 | 0.29±0.19 | 0.31±0.21 | 0.29±0.19 |
| Glutamine |  |  |  |  |  |  |
| sepsis | 330.70±114.49* | 352.10±82.69* | 408.36±166.18 | 445.92±190.57 | 415.67±157.05 | 387.79±146.28 |
| severe sepsis | 499.92±169.86* | 486.88±154.49* | 435.85±155.51 | 471.41±140.68 | 430.07±138.93 | 424.49±141.01 |
| Glutamic acid |  |  |  |  |  |  |
| sepsis | 180.30±117.44 | 189.14±100.13 | 226.05±99.93* | 180.27±65.79 | 235.44±136.40* | 219.49±133.82 |
| severe sepsis | 108.32±70.77 | 116.97±49.09 | 123.40±82.88* | 123.13±79.14 | 127.21±93.07* | 139.46±95.75 |
| Glycine |  |  |  |  |  |  |
| sepsis | 254.36±64.30 | 263.44±59.66 | 312.46±88.85 | 310.85±117.08 | 313.47±81.09 | 291.92±82.11 |
| severe sepsis | 344.88±216.78 | 283.72±66.11 | 284.03±90.78 | 282.85±78.06 | 277.14±82.42 | 272.02±82.25 |
| Homocitrulline |  |  |  |  |  |  |
| sepsis | 1.01±1.35 | 1.03±1.67 | 1.05±1.45 | 0.90±1.43 | 1.16±1.46 | 1.15±1.40 |
| severe sepsis | 1.52±2.05 | 1.56±1.72 | 1.65±1.79 | 1.50±1.87 | 1.73±2.01 | 1.77±1.93 |
| Homocystine |  |  |  |  |  |  |
| sepsis | 0.10±0.06 | 0.10±0.03 | 0.10±0.04 | 0.10±0.06 | 0.12±0.10 | 0.10±0.07 |
| severe sepsis | 0.21±0.21 | 0.17±0.17 | 0.19±0.18 | 0.18±0.20 | 0.21±0.39 | 0.16±0.21 |
| Histidine |  |  |  |  |  |  |
| sepsis | 54.54±16.56 | 54.84±15.43 | 62.14±16.57 | 61.18±18.72 | 62.62±16.01 | 61.39±16.55 |
| severe sepsis | 69.74±32.52 | 64.19±14.12 | 64.88±17.96 | 65.39±16.77 | 63.11±16.23 | 61.94±11.59 |
| δ-hydroxylysine |  |  |  |  |  |  |
| sepsis | 1.51±0.47 | 1.52±0.45 | 1.66±0.52 | 1.74±0.48 | 1.62±0.48 | 1.59±0.33 |
| severe sepsis | 1.51±0.42 | 1.63±0.38 | 1.48±0.41 | 1.60±0.49 | 1.55±0.55 | 1.65±0.52 |
| Hydroxy-L-Proline |  |  |  |  |  |  |
| sepsis | 18.61±17.62 | 17.96±16.94 | 19.31±18.95 | 20.55±15.46 | 17.45±11.90 | 16.70±8.23 |
| severe sepsis | 21.92±16.57 | 17.18±8.65 | 17.83±13.21 | 17.67±11.76 | 14.22±7.71 | 18.80±8.76 |
| Isoleucine |  |  |  |  |  |  |
| sepsis | 68.63±26.32 | 73.55±33.94 | 76.27±24.91 | 84.50±51.89 | 91.73±57.76 | 75.43±29.65 |
| severe sepsis | 67.33±30.71 | 68.59±34.22 | 69.21±32.67 | 87.80±62.30 | 54.41±19.80 | 61.28±12.59 |
| Leucine |  |  |  |  |  |  |
| sepsis | 120.18±35.90 | 131.31±44.33 | 126.83±19.17 | 147.02±67.29 | 156.22±63.83* | 142.11±50.13 |
| severe sepsis | 114.32±44.07 | 119.61±44.90 | 123.44±44.22 | 137.94±70.27 | 108.63±39.51* | 116.18±36.02 |
| Lysine |  |  |  |  |  |  |
| sepsis | 138.53±44.80 | 154.56±51.55 | 169.90±56.67 | 193.42±132.69 | 199.09±96.91 | 186.63±66.53 |
| severe sepsis | 147.21±51.86 | 162.94±64.59 | 177.19±59.92 | 176.00±60.15 | 159.34±48.52 | 160.91±49.49 |
| Methionine |  |  |  |  |  |  |
| sepsis | 14.59±8.84 | 15.81±7.09 | 19.89±10.73 | 21.15±20.34 | 19.49±13.54 | 19.32±9.79 |
| severe sepsis | 20.49±10.92 | 18.98±9.95 | 19.29±8.96 | 27.26±18.77 | 20.25±10.05 | 17.26±8.11 |
| Ornithine |  |  |  |  |  |  |
| sepsis | 75.21±32.45 | 75.71±20.08 | 86.76±31.52 | 75.24±36.38 | 99.95±53.06 | 93.87±34.26 |
| severe sepsis | 80.33±27.48 | 88.61±29.81 | 82.52±30.26 | 85.05±32.38 | 80.29±30.78 | 81.91±36.41 |
| Phosphoethanolamine |  |  |  |  |  |  |
| sepsis | 1.05±1.64 | 1.17±2.37 | 1.00±1.43 | 1.08±1.72 | 0.88±0.78 | 1.83±2.86 |
| severe sepsis | 1.28±1.26 | 0.72±0.85 | 1.22±1.07 | 1.09±1.04 | 1.01±1.22 | 1.12±1.28 |
| Phenylalanine |  |  |  |  |  |  |
| sepsis | 124.02±51.05 | 111.27±21.83 | 119.60±30.18 | 110.31±33.90 | 104.55±22.29 | 109.71±24.90* |
| severe sepsis | 110.80±40.34 | 145.71±88.05 | 127.14±56.68 | 136.53±52.42 | 130.86±54.27 | 139.05±48.84* |
| Proline |  |  |  |  |  |  |
| sepsis | 116.32±32.15* | 126.74±40.29 | 148.74±58.89 | 169.99±61.71 | 140.94±40.62 | 133.93±28.58 |
| severe sepsis | 164.25±77.97* | 146.90±49.25 | 151.32±21.32 | 144.54±51.65 | 142.75±51.22 | 150.13±40.24 |
| PhosphoSerine |  |  |  |  |  |  |
| sepsis | 1.84±2.62 | 3.12±6.42 | 2.21±3.12 | 1.70±3.03 | 1.60±1.90 | 4.88±13.42 |
| severe sepsis | 1.80±1.46 | 2.07±2.91 | 2.43±2.73 | 2.78±5.14 | 2.29±4.13 | 3.17±6.03 |
| Sarcosine |  |  |  |  |  |  |
| sepsis | 1.84±1.33 | 1.90±1.41 | 2.10±1.90 | 2.11±1.46 | 2.37±1.90 | 2.08±1.30 |
| severe sepsis | 2.91±4.22 | 3.09±2.68 | 2.96±1.84 | 3.01±1.85 | 3.19±1.77 | 3.25±1.89 |
| Serine |  |  |  |  |  |  |
| sepsis | 132.53±32.03 | 149.70±40.35 | 157.11±46.37 | 160.93±13.85 | 165.82±40.14* | 151.66±43.92* |
| severe sepsis | 121.81±57.15 | 132.19±46.76 | 124.47±51.20 | 124.59±60.97 | 112.53±43.22* | 113.15±40.10* |
| Taurine |  |  |  |  |  |  |
| sepsis | 119.91±66.15* | 128.94±61.30* | 141.52±53.66* | 129.52±50.03* | 164.50±65.56* | 157.38±49.08* |
| severe sepsis | 72.51±39.98* | 83.08±45.57* | 76.50±43.60* | 89.81±47.67* | 88.13±55.00* | 97.81±69.77* |
| Threonine |  |  |  |  |  |  |
| sepsis | 105.88±41.31 | 140.86±87.83 | 168.46±118.67 | 176.10±123.81 | 179.38±98.34 | 164.19±104.52 |
| severe sepsis | 109.25±50.28 | 122.23±63.45 | 120.03±51.39 | 126.30±55.90 | 113.25±40.27 | 110.29±35.32 |
| Tryptophan |  |  |  |  |  |  |
| sepsis | 42.33±17.42 | 45.92±16.02 | 44.26±15.15 | 44.61±19.56 | 44.87±13.14 | 50.92±17.96* |
| severe sepsis | 36.23±17.16 | 36.33±16.49 | 39.45±15.59 | 39.98±13.19 | 40.43±14.71 | 37.17±12.79* |
| Tyrosine |  |  |  |  |  |  |
| sepsis | 51.95±15.11 | 57.87±16.01 | 54.87±13.89 | 62.20±23.83 | 66.34±20.33 | 58.71±17.83 |
| severe sepsis | 53.90±19.24 | 70.23±33.79 | 64.04±21.69 | 67.13±26.62 | 65.91±30.36 | 62.35±24.46 |
| Valine |  |  |  |  |  |  |
| sepsis | 244.50±78.76 | 284.20±100.55 | 264.21±68.53 | 298.43±131.75 | 318.83±166.00 | 288.51±84.02 |
| severe sepsis | 224.28±69.06 | 267.46±118.93 | 246.18±93.22 | 274.34±113.34 | 239.41±91.96 | 239.05±74.68 |
| EAA |  |  |  |  |  |  |
| sepsis | 858.48±223.40 | 957.48±231.55 | 989.39±231.55 | 1075.52±493.61 | 1104.05±368.49* | 1028.19±287.50* |
| severe sepsis | 829.88±256.41 | 898.70±421.32 | 921.92±280.88 | 943.86±419.30 | 845.84±258.96* | 841.90±206.51* |
| NEAA |  |  |  |  |  |  |
| sepsis | 1593.91±315.83 | 1719.44±299.44 | 1993.55±456.10 | 1980.64±645.96 | 1855.29±647.00 | 1874.69±363.32 |
| severe sepsis | 1790.92±822.27 | 1714.54±534.79 | 1765.90±446.70 | 1752.75±446.70 | 1620.520±456.82 | 1637.88±491.64 |
| GAA |  |  |  |  |  |  |
| sepsis | 1801.04±375.18 | 1945.77±336.29 | 2202.89±473.48 | 2216.88±715.54 | 2086.74±743.13 | 2104.49±398.86 |
| severe sepsis | 1969.86±876.59 | 1901.40±613.98 | 1948.05±495.91 | 1948.46±631.67 | 1784.75±517.95 | 1805.28±539.34 |
| BCAA |  |  |  |  |  |  |
| sepsis | 433.31±136.06 | 489.06±175.20 | 467.32±106.40 | 529.94±247.93 | 558.44±286.28* | 499.20±160.27* |
| severe sepsis | 405.92±137.50 | 455.66±191.53 | 438.82±157.81 | 494.92±234.83 | 387.33±134.04* | 385.88±110.91* |
| AAA |  |  |  |  |  |  |
| sepsis | 218.30±75.04 | 215.06±38.52 | 218.73±39.23 | 217.12±66.21 | 215.77±31.12 | 219.33±38.51 |
| severe sepsis | 200.92±59.45 | 252.26±126.16 | 230.63±79.43 | 243.63±77.13 | 237.20±88.57 | 238.54±74.24 |
| SAA |  |  |  |  |  |  |
| sepsis | 155.01±67.98 | 164.91±79.68 | 180.77±79.49* | 175.83±79.64 | 221.06±50.55* | 213.73±46.02* |
| severe sepsis | 121.09±42.46 | 127.48±54.82 | 123.16±46.73* | 141.84±43.38 | 129.38±60.64* | 133.51±73.02* |
| BCAA/AAA |  |  |  |  |  |  |
| sepsis | 2.07±0.70 | 2.32±0.99 | 2.19±0.67 | 2.45±0.89 | 2.63±1.53* | 2.25±0.46* |
| severe sepsis | 2.06±0.51 | 1.91±0.51 | 2.00±0.68 | 2.04±0.78 | 1.69±0.37* | 1.66±0.40* |

AA, amino acids; EAA, essential AA; NEAA, nonessential AA; GAA, glycogenic AA; BCAA, branched-chain AA; AAA, aromatic AA; SAA, sulfur-containing AA

* indicates severe sepsis vs. sepsis, P<0.05
