# Supplementary material for: Dynamic Changes in Amino Acid Concentration Profiles in Patients with Sepsis
Source: PLoS One. 2015 Apr 7;10(4):e0121933. doi: 10.1371/journal.pone.0121933 (PMC4388841; doi:10.1371/journal.pone.0121933)
Supplement: S4 Table — (DOC) [file pone.0121933.s004.doc]

S4 Table Comparison of amino acid serum concentrations between survivor (20 cases) and non-survivor (15 cases) groups.

| **Amino acid** | **Day 1** | **Day 3** | **Day 5** | **Day 7** | **Day 10** | **Day 14** |
| --- | --- | --- | --- | --- | --- | --- |
| 1-Methyl-L-Histidine |  |  |  |  |  |  |
| survivor | 2.32±1.23 | 2.76±2.05 | 2.45±1.07 | 2.76±1.24 | 2.86±1.64 | 3.58±2.11 |
| non-survivor | 2.56±1.46 | 2.34±0.81 | 2.40±1.11 | 1.99±0.65 | 2.14±1.20 | 2.29±1.32 |
| 3-Methyl-L-Histidine |  |  |  |  |  |  |
| survivor | 4.74±4.55 | 5.46±4.94 | 5.56±5.40 | 5.02±5.61 | 5.16±6.45 | 5.15±5.69 |
| non-survivor | 6.64±5.97 | 7.56±5.77 | 8.25±6.51 | 7.43±4.62 | 7.73±4.13 | 7.25±4.72 |
| α-aminoadipic acid |  |  |  |  |  |  |
| survivor | 1.29±0.46* | 1.53±0.82 | 1.56±0.92 | 1.74±0.69 | 1.38±0.65* | 1.33±0.37* |
| non-survivor | 2.01±0.69* | 2.40±1.49 | 2.57±2.07 | 2.62±2.19 | 2.48±1.76* | 2.42±1.71* |
| α-amino-n-butyric acid |  |  |  |  |  |  |
| survivor | 14.67±9.93 | 16.17±8.97 | 14.22±7.21 | 18.48±16.37 | 14.24±9.90 | 15.07±10.05 |
| non-survivor | 18.35±13.15 | 18.51±15.35 | 19.11±18.22 | 23.42±25.79 | 23.68±25.70 | 20.86±23.04 |
| Alanine |  |  |  |  |  |  |
| survivor | 315.30±199.13 | 304.63±102.61 | 329.43±138.13 | 332.13±143.70 | 312.68±96.18 | 340.56±97.95 |
| non-survivor | 361.97±185.77 | 333.23±112.25 | 353.37±108.57 | 343.55±115.78 | 359.46±112.41 | 352.95±132.83 |
| Anserine |  |  |  |  |  |  |
| survivor | 0.14±0.04 | 0.17±0.06 | 0.17±0.07 | 0.20±0.07 | 0.16±0.06 | 0.19±0.06 |
| non-survivor | 0.17±0.06 | 0.16±0.04 | 0.17±0.07 | 0.15±0.06 | 0.15±0.08 | 0.19±0.07 |
| Arginine |  |  |  |  |  |  |
| survivor | 72.04±27.65 | 73.00±28.15 | 78.38±40.41 | 114.93±65.81* | 88.56±38.17 | 86.42±25.08* |
| non-survivor | 61.17±28.57 | 74.60±28.76 | 78.87±24.69 | 69.27±17.66* | 71.57±25.20 | 64.51±17.56* |
| Argininosuccinic acid |  |  |  |  |  |  |
| survivor | 0.10±0.07 | 0.12±0.01 | 0.13±0.15 | 0.11±0.08 | 0.12±0.08 | 0.12±0.10 |
| non-survivor | 0.14±0.09 | 0.19±0.15 | 0.14±0.08 | 0.14±0.11 | 0.15±0.09 | 0.163±0.10 |
| Asparagine |  |  |  |  |  |  |
| survivor | 48.67±20.85 | 56.02±19.12 | 61.29±26.44 | 72.83±39.55 | 61.78±21.49 | 60.33±22.89 |
| non-survivor | 54.40±14.82 | 54.85±21.75 | 63.29±20.13 | 61.77±17.94 | 58.92±15.93 | 60.16±16.71 |
| Aspartic acid |  |  |  |  |  |  |
| survivor | 39.28±17.02 | 38.31±15.73 | 39.23±24.19 | 39.59±19.00 | 42.97±24.53 | 45.50±20.31 |
| non-survivor | 38.22±27.29 | 40.03±24.32 | 42.22±31.97 | 31.33±19.22 | 30.40±17.84 | 32.50±20.52 |
| β-aminoisobutyric acid |  |  |  |  |  |  |
| survivor | 5.78±11.50 | 7.04±14.39 | 6.53±14.81 | 2.76±4.22 | 3.11±5.90 | 3.35±4.60 |
| non-survivor | 9.13±11.18 | 8.59±16.74 | 46.90±36.20 | 12.68±27.43 | 8.91±17.96 | 8.37±16.42 |
| β-Alanine |  |  |  |  |  |  |
| survivor | 17.74±5.39 | 18.08±4.95 | 17.92±5.57 | 16.40±4.76 | 17.01±5.90 | 18.03±2.74 |
| non-survivor | 20.71±3.88 | 20.01±3.18 | 16.74±4.52 | 15.67±4.01 | 16.74±3.57 | 17.35±4.06 |
| carnosine |  |  |  |  |  |  |
| survivor | 0.08±0.03 | 0.11±0.07 | 0.07±0.03 | 0.07±0.04 | 0.08±0.03 | 0.09±0.03 |
| non-survivor | 0.07±0.03 | 0.10±0.03 | 0.09±0.03 | 0.10±0.06 | 0.10±0.04 | 0.09±0.04 |
| Citrulline |  |  |  |  |  |  |
| survivor | 14.60±7.07 | 19.13±7.30 | 17.81±7.74 | 19.28±6.53 | 20.40±10.91 | 19.92±8.26 |
| non-survivor | 14.45±7.29 | 14.34±7.63 | 17.15±10.81 | 15.46±8.93 | 14.88±7.08 | 14.60±6.65 |
| Cystathionine |  |  |  |  |  |  |
| survivor | 3.09±1.17* | 3.63±1.20 | 3.63±1.36 | 3.95±1.11 | 3.46±1.19* | 3.39±0.49* |
| non-survivor | 4.12±1.17* | 4.17±1.02 | 4.42±1.85 | 4.42±1.42 | 4.93±1.48* | 4.61±1.65* |
| Cystine |  |  |  |  |  |  |
| survivor | 22.19±12.16 | 31.33±12.86 | 32.90±19.14 | 38.53±22.62 | 32.39±15.77 | 34.71±11.42 |
| non-survivor | 20.67±8.82 | 26.13±17.83 | 27.50±22.76 | 26.49±14.75 | 29.23±14.48 | 28.27±15.29 |
| Ethanolamine |  |  |  |  |  |  |
| survivor | 9.22±2.70* | 11.50±4.36 | 10.95±3.69 | 10.91±5.16 | 12.05±5.06 | 11.37±4.26 |
| non-survivor | 13.66±4.63* | 14.76±6.59 | 14.51±7.74 | 14.16±4.03 | 13.82±3.86 | 13.85±4.85 |
| γ-Amino-n-Butyric Acid |  |  |  |  |  |  |
| survivor | 0.25±0.14 | 0.34±0.19 | 0.26±0.19 | 0.27±0.16 | 0.28±0.23 | 0.33±0.26 |
| non-survivor | 0.39±0.31 | 0.29±0.18 | 0.29±0.17 | 0.31±0.18 | 0.31±0.22 | 0.29±0.20 |
| Glutamine |  |  |  |  |  |  |
| survivor | 416.23±179.49 | 453.01±149.06 | 442.78±149.05 | 484.53±175.76 | 428.36±148.76 | 417.47±146.51 |
| non-survivor | 447.69±158.95 | 416.70±148.25 | 407.81±169.27 | 440.08±139.24 | 420.13±141.71 | 403.65±141.98 |
| Glutamic acid |  |  |  |  |  |  |
| survivor | 141.46±108.76 | 143.01±88.73 | 162.04±96.14 | 176.76±90.58* | 195.66±132.07 | 202.15±123.01 |
| non-survivor | 134.47±83.79 | 142.34±62.21 | 149.79±107.27 | 114.14±51.76* | 129.45±96.93 | 139.64±104.76 |
| Glycine |  |  |  |  |  |  |
| survivor | 252.95±100.33 | 262.72±66.16 | 287.33±99.72 | 313.76±113.15 | 304.79±87.82 | 291.98±69.62 |
| non-survivor | 392.41±231.08 | 297.72±55.47 | 300.33±77.35 | 274.14±68.84 | 273.57±74.40 | 266.66±92.31 |
| Homocitrulline |  |  |  |  |  |  |
| survivor | 1.09±1.30 | 1.18±1.43 | 1.24±1.39 | 1.00±1.51 | 1.31±1.74 | 1.50±1.69 |
| non-survivor | 1.63±2.39 | 1.67±2.07 | 1.71±2.01 | 1.57±1.92 | 1.80±1.96 | 1.55±1.84 |
| Homocystine |  |  |  |  |  |  |
| survivor | 0.14±0.12 | 0.16±0.17 | 0.17±0.19 | 0.16±0.23 | 0.22±0.40 | 0.16±0.25 |
| non-survivor | 0.20±0.23 | 0.12±0.07 | 0.14±0.10 | 0.13±0.07 | 0.12±0.05 | 0.11±0.05 |
| Histidine |  |  |  |  |  |  |
| survivor | 62.34±26.11 | 61.53±16.21 | 63.81±18.09 | 65.43±18.42 | 63.96±17.85 | 61.87±14.54 |
| non-survivor | 64.86±31.19 | 59.80±13.67 | 64.22±16.94 | 62.34±16.71 | 61.45±13.13 | 61.61±12.75 |
| δ-hydroxylysine |  |  |  |  |  |  |
| survivor | 1.60±0.41 | 1.61±0.38 | 1.57±0.43 | 1.74±0.42 | 1.63±0.49 | 1.68±0.33 |
| non-survivor | 1.36±0.44 | 1.56±0.45 | 1.49±0.48 | 1.57±0.54 | 1.50±0.57 | 1.58±0.55 |
| Hydroxy-L-Proline |  |  |  |  |  |  |
| survivor | 18.73±14.97 | 16.82±13.20 | 16.65±14.86 | 18.38±13.42 | 15.91±10.58 | 17.26±7.03 |
| non-survivor | 23.38±19.80 | 18.45±10.26 | 20.33±15.45 | 19.03±13.04 | 14.93±8.26 | 18.70±9.84 |
| Isoleucine |  |  |  |  |  |  |
| survivor | 72.19±32.51 | 69.30±30.73 | 68.45±25.18 | 85.22±50.24 | 74.46±49.63 | 71.93±26.29 |
| non-survivor | 60.98±19.77 | 72.00±39.09 | 75.17±35.92 | 87.85±65.95 | 59.82±21.18 | 59.76±12.25 |
| Leucine |  |  |  |  |  |  |
| survivor | 121.38±47.84 | 125.19±41.57 | 119.05±30.77 | 148.27±65.02 | 132.06±63.14 | 132.30±49.46 |
| non-survivor | 109.48±26.03 | 121.62±50.11 | 131.19±44.91 | 134.84±72.49 | 119.06±40.18 | 120.16±36.76 |
| Lysine |  |  |  |  |  |  |
| survivor | 153.66±53.29 | 158.87±50.31 | 161.58±60.51 | 206.44±120.94 | 179.98±84.12 | 183.20±66.94 |
| non-survivor | 127.14±35.43 | 161.60±73.98 | 190.93±52.53 | 160.16±46.66 | 166.54±52.43 | 158.96±44.66 |
| Methionine |  |  |  |  |  |  |
| survivor | 17.76±11.21 | 16.84±7.43 | 18.98±9.91 | 22.03±18.09 | 19.63±12.14 | 18.06±9.13 |
| non-survivor | 18.36±9.33 | 19.40±11.29 | 20.08±9.04 | 22.74±20.62 | 10.76±10.00 | 19.15±8.95 |
| Ornithine |  |  |  |  |  |  |
| survivor | 76.64±31.59 | 81.89±28.36 | 82.36±29.73 | 83.20±40.10 | 91.18±47.54 | 90.91±33.49 |
| non-survivor | 80.61±26.25 | 87.27±25.96 | 85.75±31.80 | 79.77±27.62 | 82.88±30.52 | 82.27±37.92 |
| Phosphoethanolamine |  |  |  |  |  |  |
| survivor | 1.51±1.67 | 1.15±1.91 | 1.37±1.39 | 1.39±1.65 | 1.24±1.24 | 2.11±2.66 |
| non-survivor | 0.67±0.61 | 0.46±0.47 | 0.90±0.83 | 0.81±0.83 | 0.57±0.56 | 0.72±0.73 |
| Phenylalanine |  |  |  |  |  |  |
| survivor | 115.58±47.85 | 113.81±37.66 | 108.82±31.10 | 105.66±36.96* | 104.96±34.50* | 114.75±26.39 |
| non-survivor | 117.69±41.61 | 163.69±102.17 | 144.01±60.68 | 146.46±48.85* | 143.44±52.48* | 140.21±52.70 |
| Proline |  |  |  |  |  |  |
| survivor | 127.47±49.03 | 132.67±40.07 | 146.04±52.85 | 166.54±52.77 | 141.34±51.08 | 143.85±37.08 |
| non-survivor | 170.37±83.43 | 150.56±55.35 | 155.89±54.43 | 142.29±47.01 | 143.14±41.94 | 144.11±37.42 |
| PhosphoSerine |  |  |  |  |  |  |
| survivor | 2.34±2.37* | 3.35±5.47 | 2.86±3.05 | 3.92±6.06 | 2.55±4.31 | 6.39±13.05 |
| non-survivor | 0.99±0.58* | 1.04±0.91 | 1.76±2.46 | 0.95±0.93 | 1.29±1.41 | 1.42±1.84 |
| Sarcosine |  |  |  |  |  |  |
| survivor | 1.83±1.32 | 2.72±2.77 | 2.55±1.85 | 2.40±1.38 | 2.45±1.61 | 2.46±1.60 |
| non-survivor | 3.47±5.07 | 2.58±1.63 | 2.84±1.96 | 2.93±2.05 | 3.50±2.01 | 3.13±1.89 |
| Serine |  |  |  |  |  |  |
| survivor | 129.78±49.39 | 149.35±45.84 | 145.92±58.31 | 171.61±75.21* | 150.30±52.47* | 148.46±44.36* |
| non-survivor | 120.86±46.71 | 121.59±38.83 | 121.74±39.33 | 106.89±25.38* | 109.17±31.76* | 108.22±37.18* |
| Taurine |  |  |  |  |  |  |
| survivor | 101.88±52.96 | 103.59±56.93 | 107.23±56.38 | 119.77±40.98 | 140.75±72.40* | 148.40±58.78* |
| non-survivor | 76.51±57.42 | 93.07±54.81 | 85.62±54.15 | 90.36±57.40 | 83.59±49.89* | 94.27±68.11* |
| Threonine |  |  |  |  |  |  |
| survivor | 114.88±52.17 | 135.86±76.14 | 149.67±102.14 | 180.51±112.96 | 149.22±87.86 | 150.58±95.67 |
| non-survivor | 96.54±32.73 | 118.44±66.94 | 118.62±40.32 | 111.54±37.60 | 122.92±48.39 | 112.21±38.39 |
| Tryptophan |  |  |  |  |  |  |
| survivor | 41.44±19.02 | 44.72±16.52* | 44.21±15.93 | 45.75±17.98 | 45.10±15.47 | 49.04±17.87* |
| non-survivor | 34.60±13.67 | 32.09±14.47* | 37.11±14.25 | 37.92±12.61 | 37.88±11.06 | 36.13±11.78* |
| Tyrosine |  |  |  |  |  |  |
| survivor | 52.42±19.54 | 64.09±23.66 | 59.26±17.39 | 66.62±2457 | 63.99±26.22 | 56.83±17.03 |
| non-survivor | 54.11±13.94 | 68.48±63.89 | 63.29±22.80 | 64.08±26.76 | 69.03±28.01 | 64.84±25.65 |
| Valine |  |  |  |  |  |  |
| survivor | 242.42±84.29 | 282.88±100.12 | 265.82±85.35 | 311.34±132.35 | 283.62±152.88 | 272.42±85.82 |
| non-survivor | 217.49±48.29 | 258.86±129.65 | 235.22±85.27 | 257.19±102.07 | 249.57±85.43 | 244.18±75.87 |
| EAA |  |  |  |  |  |  |
| survivor | 879.30±277.22 | 947.47±257.17 | 936.56±276.32 | 1026.32±546.25 | 989.02±378.13 | 992.28±278.95 |
| non-survivor | 782.27±154.21 | 878.59±486.99 | 952.31±258.35 | 955.44±325.68 | 879.70±229.87 | 838.16±207.87 |
| NEAA |  |  |  |  |  |  |
| survivor | 1680.14±606.22 | 1769.66±329.62 | 1848.41±509.88 | 1931.92±778.90 | 1773.36±602.27 | 1745.84±564.30 |
| non-survivor | 1755.05±764.40 | 1640.53±610.47 | 1828.32±397.24 | 1736.37±285.55 | 1625.14±450.49 | 1703.58±337.02 |
| GAA |  |  |  |  |  |  |
| survivor | 1887.89±659.91 | 1988.45±375.78 | 2054.96±557.26 | 2159.16±878.80 | 1980.79±695.93 | 1947.06±633.38 |
| non-survivor | 1920.27±811.19 | 1815.04±697.09 | 2000.25±428.27 | 1929.48±333.43 | 1791.80±505.98 | 1882.93±358.91 |
| BCAA |  |  |  |  |  |  |
| survivor | 435.98±158.19 | 477.37±165.64 | 453.32±134.5 | 544.82±242.84 | 490.14±260.56 | 476.64±155.17 |
| non-survivor | 387.95±85.00 | 452.47±215.39 | 441.58±156.02 | 473.60±232.28 | 398.54±127.78 | 384.26±113.02 |
| AAA |  |  |  |  |  |  |
| survivor | 209.45±72.50 | 226.62±60.90 | 212.30±49.52 | 218.03±66.99 | 214.05±65.65 | 220.62±39.79 |
| non-survivor | 206.40±56.58 | 264.25±148.97 | 244.40±84.97 | 248.46±77.94 | 250.35±78.59 | 241.18±79.01 |
| SAA |  |  |  |  |  |  |
| survivor | 141.97±58.44 | 151.93±59.69 | 159.28±63.42 | 180.54±54.05* | 192.98±72.93* | 201.33±62.26* |
| non-survivor | 125.00±53.49 | 127.16±75.57 | 124.46±63.70 | 133.23±60.12* | 123.31±48.80 | 129.04±69.45* |
| BCAA/AAA |  |  |  |  |  |  |
| survivor | 2.15±0.66 | 2.22±0.81 | 2.21±0.70 | 2.48±0.79 | 2.35±1.28 | 2.13±0.46* |
| non-survivor | 1.93±0.43 | 1.80±0.50 | 1.88±0.61 | 1.93±1.80 | 1.62±0.41 | 1.62±0.45* |

AA, amino acids; EAA, essential AA; NEAA, nonessential AA; GAA, glycogenic AA; BCAA, branched-chain AA; AAA, aromatic AA; SAA, sulfur-containing AA

* indicates non-survivor vs. survivor, P<0.05
